# Appearance anxiety and social anxiety: A mediated model of self-compassion

**DOI:** 10.3389/fpubh.2023.1105428

**Published:** 2023-03-21

**Authors:** Jie Gao, Yi Feng, Shicun Xu, Amanda Wilson, Hui Li, Xiaofeng Wang, Xi Sun, Yuanyuan Wang

**Affiliations:** ^1^Key Laboratory of Brain, Cognition and Education Sciences, Ministry of Education, Guangzhou, China; ^2^School of Psychology, Center for Studies of Psychological Application, and Guangdong Key Laboratory of Mental Health and Cognitive Science, South China Normal University, Guangzhou, China; ^3^Mental Health Center, Central University of Finance and Economics, Beijing, China; ^4^Faculty of Psychology, Beijing Normal University, Beijing, China; ^5^Northeast Asian Research Center, Jilin University, Changchun, China; ^6^Department of Population, Resources and Environment, Northeast Asian Studies College, Jilin University, Changchun, China; ^7^China Center for Aging Studies and Social-Economic Development, Jilin University, Changchun, China; ^8^Division of Psychology, Faculty of Health and Life Sciences, De Montfort University, Leicester, United Kingdom; ^9^School of Public Health, Jilin University, Changchun, China

**Keywords:** appearance anxiety, social anxiety, self-compassion, mediating effect, structural equation model (SEM)

## Abstract

**Background:**

Previous studies have focused on the comorbidity of appearance anxiety and social anxiety, but few studies have focused on the protective role of self-compassion as underlying this mechanism, in young people like University students. With the increase of prevalence of appearance anxiety and social anxiety in this age group, it is necessary to explore factors that can buffer against the symptoms of these disorders. Therefore, the aims of this study were to research the effect of appearance anxiety and social anxiety, then to examine whether self-compassion has a protective effect on social anxiety.

**Method:**

The study was cross-sectional and conducted online from October 2021 to November 2021 in Jilin Province, China. A total of 63 Universities in the province participated in this study, totaling 96,218 participants, of which 40,065 were males (41.64%) and 56,153 females (58.36%), the mean age of the sample was 19.59 (±1.74). The Appearance Anxiety Scale-Brief Version was used to measure appearance anxiety. The Social Anxiety subscale of the Self-Consciousness Scale was used to measure social anxiety. The Self-Compassion Scale-Short Form was used to measure self-compassion. A structural equation model (SEM) was run to examine the mediating effect of self-compassion on the relationship between appearance anxiety and social anxiety.

**Result:**

Overall, appearance anxiety was positively associated with social anxiety [β = 0.334, 95% CI = (0.328, 0.341), *p* < 0.001], and self-compassion could mediate the effect of appearance anxiety on social anxiety [β = 0.128, 95% CI = (0.124, 0.132), *p* < 0.001]. Self-compassion played a partial mediating role between appearance anxiety and social anxiety.

**Conclusion:**

Individuals with high appearance anxiety are also at higher risk of social anxiety, but self-compassion can buffer against this relationship. These findings begin to explore novel approaches to treat social anxiety and can provide valuable insights for self-compassion training.

## 1. Introduction

Appearance anxiety is a subclinical indicator of body dysmorphic disorder, which usually manifests as excessive anxiety about certain physical defects that are often perceived as normal by others (1). As the main users of various social media, University students are overexposed to excessive attention and comparison of their appearance, thus increasing the risk of appearance anxiety (2). The campus media of China Youth Daily conducted a questionnaire survey on the topic of appearance anxiety among 2,063 University students nationwide (3). The results showed that 59.03% of University students were anxious about their appearance. Furthermore, only a small percentage (7.87%) were very satisfied with their appearance, 9.66% were not very satisfied with their appearance, and 2.45% were very dissatisfied. Previous studies have found that many psychiatric disorders, including body dysmorphic disorder (4), anorexia nervosa, bulimia nervosa (5), pathological narcissism (6), and social anxiety (7), are all associated with high attention or negative perception of appearance. Therefore, it is important to investigate the appearance anxiety of students and the influencing factors.

Among the range of psychiatric disorders, social anxiety is widely regarded within the literature as most related to appearance anxiety. For example, studies have found that dissatisfaction with appearance can significantly affect social interactions (8) and increase the risk of social anxiety (9) or other psychiatric problems (10). Appearance anxiety is not limited to dissatisfaction with weight and appearance, but also negative perceptions of height can lead to social anxiety (11). Appearance anxiety may also increase the risk of dating anxiety and dieting behaviors (12) in young adults who are affected by social anxiety (13). Therefore, while the relationship between appearance anxiety and social anxiety has been well-established, it is important to begin to explore the relationship between appearance anxiety and social anxiety, as well as begin to identify variables that may influence this effect.

As one of the positive psychological qualities that have received widespread attention in recent years, self-compassion has been revealed to affect a range of mental health problems, including anxiety (14, 15), depression (16) and stress (17). Self-compassion refers to a non-judgmental, kind and tolerant attitude toward one's own pain and deficiency (18). Appearance anxiety was found to be positively correlated with the negative sense component of self-compassion (19). Therefore, low levels of self-compassion may increase the risk of social anxiety (20). In addition, studies have found self-compassion to be an important factor in alleviating anxiety and depression symptoms (21) and it could also have played a positive role during the COVID-19 pandemic (22). Meanwhile, self-compassion may mediate the relationship between perfectionism and social anxiety, which may be protective for individuals experiencing poor body image and the associated maladaptive outcomes (23). Therefore, it is reasonable to speculate that self-compassion may mediate the effect of appearance anxiety on social anxiety.

This study aimed to examine whether appearance anxiety can lead to social anxiety and to explore the underlying mechanism of the relationship between them. The first step is to verify the direct predictive effect of appearance anxiety on social anxiety. Then, the second step is to examine the mediating effect of self-compassion between appearance anxiety and social anxiety. Combining the above procedures with the literature reviewed, we propose two hypotheses: (1) appearance anxiety will be positively associated with social anxiety; (2) self-compassion will partially mediate the effect of appearance anxiety on social anxiety.

## 2. Methods

### 2.1. Participants

This was a cross-sectional study conducted online from October 2021 to November 2021 in Jilin Province, China. Using a Quick Response Code (QR Code), students provided electronic informed consent online to complete the questionnaire. The inclusion criteria included: age 16 years old or above, studying in Universities in Jilin Province, China, and fluency to understand the Chinese assessment materials. This study was approved by the Ethics Committee of Jilin University.

### 2.2. Measures

#### 2.2.1. Social–demographic characteristics

The survey consisted of a series of scales and demographic information. The demographic measures including age, sex, ethnic group, only child or siblings, residence, and family structure. Age required participants to fill in their specific age on the horizontal line. Sex was measured by a question in which responses were categorized as male or female. Only child was measured by a single question, with responses classified as yes or no. Residence was measured by a single question, and responses were categorized as urban or rural. Finally, family structure was measured by a question: “What is your family category?”. And responses were categorized into core family, large family, foster family, reconstituted family, single parent family, and left-behind family, for a total of six levels. With the exception of age, all other social demographic questions were completed by selecting the item that best corresponded.

#### 2.2.2. Appearance anxiety

The Appearance Anxiety Scale-Brief Version was designed by Keelan et al. (24) to measure the degree to which individuals worry about their appearance. It contains 14 items rated on a five-point Likert scale from 1 (never) to 5 (always). The items of the scale include positive subscale items (e.g., I am very satisfied with my appearance) and negative subscale items (e.g., I was nervous about my appearance). Higher scores indicate a higher degree of appearance anxiety. The internal consistency coefficient of the scale reached the acceptable range (α = 0.86).

#### 2.2.3. Social anxiety

The Social Anxiety subscale of the Self-Consciousness Scale (SASS) was designed by Fenigstein and revised by Scheier and Carver (25) to measure the level of social anxiety. The scale consists of 6 items, 5 items are scored in a forward direction (e.g., I'm very easily embarrassed) and 1 item is scored in the reverse direction (e.g., It's easy for me to talk to strangers). Every item is rated on a five-point scale from 1 (strongly disagree) to 5 (totally agree). Higher scores indicate a higher degree of social anxiety. The internal consistency coefficient of the scale reached the acceptable range (α = 0.82).

#### 2.2.4. Self-compassion

In measuring the scores of self-compassion, we used the scale designed by Raes et al. (26). The scale contains 12 items rated on a five-point scale from 1 to 5. The items of the scale include positive subscale items (e.g., Understanding of aspects I do not like) and negative subscale items (e.g., Other people happier than I am). The scale is divided into six dimensions: Self-Kindness Items (2, 6), Self-Judgment Items (11, 12), Common Humanity Items (5, 10), Isolation Items (4, 8), Mindfulness Items (3, 7), Over-identified Items (1, 9). Higher scores indicate a higher degree of self-compassion. According to the reliability analysis, the internal consistency coefficient of the scale reached the acceptable range (α = 0.70).

### 2.3. Data analysis

All data analysis was conducted using IBM SPSS statistics 25.0 and Mplus 8.0. Descriptive statistical analysis was used to calculate the mean value, standard deviation and correlation coefficients of all variables. The degree of the model can be judged by the fitting index of output, including RMSEA, CFI, TLI, and SRMR. A mediated model was built utilizing path analysis. The 5,000 bootstrap method and 95% confidence interval were applied for validation of path effects (27). All significance values were set at *p* < 0.05 by two-tailed test.

## 3. Result

### 3.1. Demographic characteristics and correlation

The total sample size was 96,218, including 40,065 males (41.64%) and 56,153 females (58.36%). The average age of the sample was 19.59 (±1.74). In addition, 89.50% of them were Han nationality, 52.55% had siblings, 50.86% originally lived in urban cities, and 69.52% belonged to core families. Table 1 lists the mean values and standard deviation of all descriptive variables.

**Table 1:** Socio-demographic characteristics of the study sample (*N* = 96,218).

**Variables**	***N*/Mean**	**%/*SD***
Age	19.59	1.74
**Sex**		
Male	40,065	41.64
Female	56,153	58.36
**Only child**		
Yes	45,660	47.45
No	50,558	52.55
**Residence**		
Urban	48,932	50.86
Rural	47,286	49.14
**Ethnic group**		
Han	86,111	89.50
Others	10,107	10.50
**Family structure**		
Core	66,888	69.52
Large	17,622	18.31
Foster	135	0.14
Reconstituted	2,626	2.73
Single parent	7,529	7.82
Left-behind	1,418	1.47
**Measured variables**		
Self-compassion	39.87	6.16
Appearance anxiety	38.71	9.42
Social anxiety	11.08	5.48

**Figure 1:**
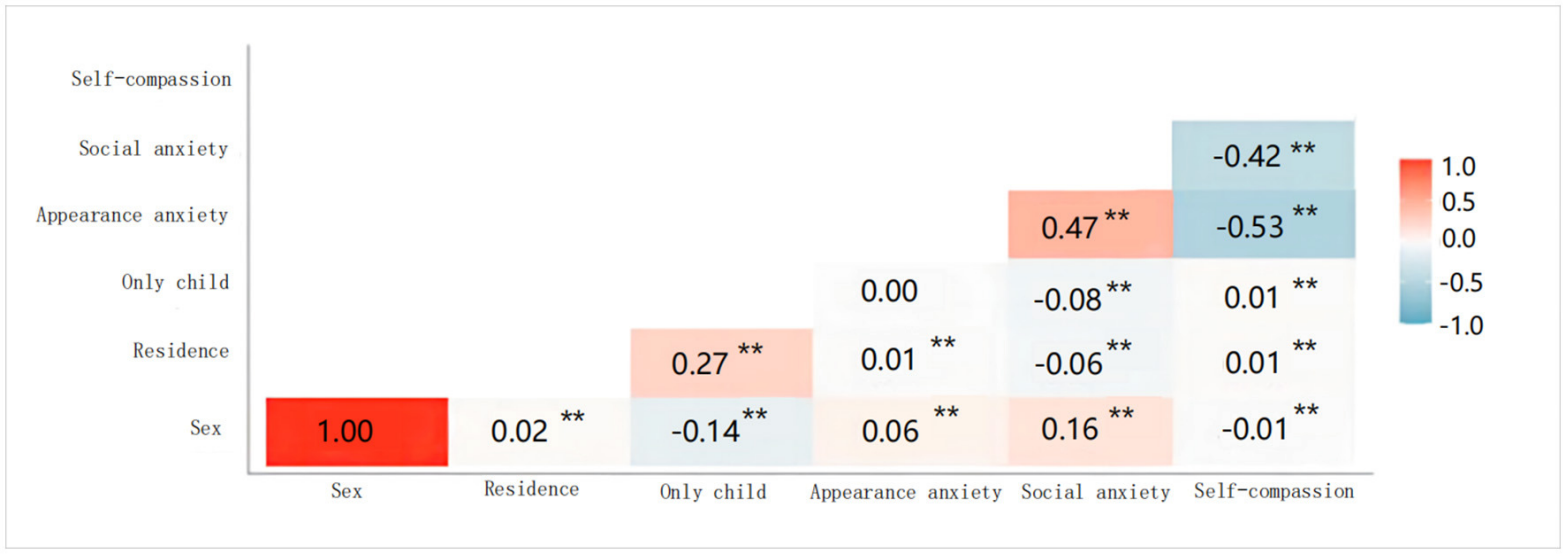
Pearson correlations of main variances. Sex, only child, and residence are dummy variables. Female = 1, others = 0; only child = 1, others = 0; urban = 1, others = 0. **p < 0.01.

Figure 1 shows the results of the Pearson correlations of the main variables. According to the result, self-compassion was negatively correlated with appearance anxiety (r = −0.534, *p* < 0.01) and social anxiety (r = −0.422, *p* < 0.01); appearance anxiety was positively correlated with social anxiety (r = 0.471, *p* < 0.01). In addition, some demographic variables that might affect the model were treated as dummy variables (0 or 1) and their correlation coefficients with the main variables were calculated.

### 3.2. The mediating role of self-compassion

**Figure 2:**
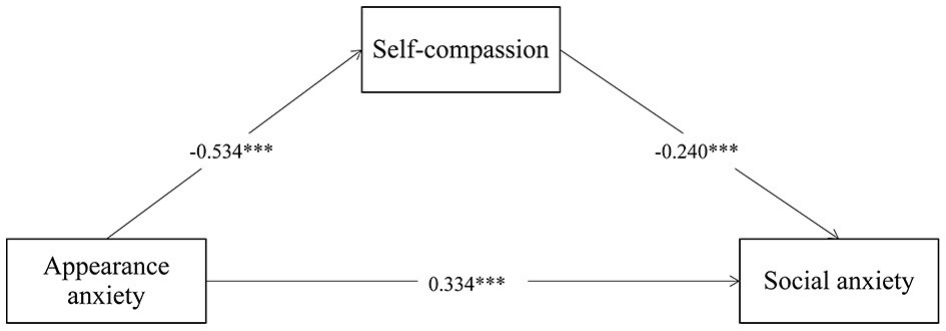
The mediated model. ***p < 0.001.

In order to ensure the accuracy of the results, the three variables of sex, only child and residence were set as covariates in our structural equation model (SEM). In addition, the fitting index of the model (Figure 2) reached an acceptable range (RMSEA = 0.016, CFI = 0.999, TLI = 0.996, SRMR = 0.007).

Appearance anxiety significantly predicted social anxiety [β = 0.334, 95% CI = (0.328, 0.341), *p* < 0.001] and this effect remained significant after adding self-compassion as a mediating variable [β = 0.128, 95% CI = (0.124, 0.132), *p* < 0.001]. The effect between appearance anxiety and self-compassion was significant [β = −0.534, 95% CI = (−0.539, −0.529), *p* < 0.001], as was the effect between self-compassion and social anxiety [β = −0.240, 95% CI = (−0.247, −0.234), *p* < 0.001]. These results indicate that self-compassion plays a mediating role between appearance anxiety on social anxiety, and only plays a partial mediating role. In terms of proportion, the direct effect was 0.334, accounting for 72.29% of the total effect, and the indirect effect was 0.128, accounting for 27.71% of the total effect.

## 4. Discussion

This study is the first to reveal the mediating role of self-compassion as well as the underlying mechanism of appearance anxiety to social anxiety. The result showed that appearance anxiety positively predicted social anxiety, and the relationship was partly achieved by effecting the level of self-compassion.

Similar to previous studies on appearance anxiety, our results found that there was a significant correlation between appearance anxiety and social anxiety, and appearance anxiety positively predicted social anxiety (28), validating hypothesis 1. As for the explanation of this correlation, different scholars have put forward different arguments. Some scholars believe that fear of negative evaluation mediates the link between appearance anxiety and social anxiety (29), and other scholars believe that from the etiology perspective social anxiety originates from individual sensitivity to anxiety (30). In addition, previous studies have also found the mediating effect of perfectionism (31), self-esteem (32), social rejection sensitivity (33), and eating disorders (34) also exist between appearance anxiety and social anxiety. Which is a point for future inquiry.

Inconsistent with previous studies, this study used self-compassion as a mediating variable between appearance anxiety and social anxiety. Several previous studies have demonstrated the positive effects of self-compassion on mental health, including that self-compassion played a mediating role on the relationship between stress and psychopathologies among Japanese workers (35). Another study further found that self-compassion has a positive effect on alleviating Shame-Proneness in social anxiety (36). In terms of the treatment of social anxiety, self-compassion training also had a positive effect (15). Self-compassion has been shown to be a lasting and stable state (37), and it is considered one of the core variables of mental health and psychopathology symptom remission (38). Therefore, on the basis of previous studies, we verified that self-compassion can play a mediating role in the mechanism of appearance anxiety for social anxiety, confirming hypothesis 2. One possible explanation for this mediating effect is that some people judge themselves by the demanding the standards of the outside world be applied to their self-image (32), which decreases their level of self-compassion. After the level of self-compassion is reduced, they cannot treat themselves with tolerance and understanding, and are afraid of being compared to others, leading to anxiety and fear of interpersonal interaction.

Previous research uses Self-objectification Theory (39) to explain appearance anxiety. The theory proposes that the cause of appearance anxiety is due to the internalization of external standards onto their own evaluation standards, leading to decreased self-esteem and mental health problems, such as anxiety and depression. Based on this theory, this study further verified and enriched this theory by using the positive prediction of appearance anxiety on social anxiety and the negative prediction of self-compassion. Notably, in contrast to self-esteem, self-compassion has no negative costs or consequences and is associated with positive mental health outcomes (40). Appearance anxiety can lead to a negative evaluation of oneself, resulting in a continuous decrease in self-compassion levels and a state of social anxiety. This paper suggests that self-compassion can also be used as a protective factor to buffer against the pathway from appearance anxiety to other negative psychological states.

In terms of other practical applications, self-compassion can be regarded as a buffer to cope with negative events. A high degree of self-compassion helps people to maintain a balanced and accepting attitude in the face of traumatic or stressful events and to avoid development of more serious psychiatric conditions. Therefore, whether it is in the process of treatment or preventing mental health problems, through routine training, an individual can make full use of the positive role of self-compassion. And it may be practical to adopt methods like mindfulness meditation training, leading the individual to have more tolerance and an understanding attitude to improve their ability to cope with stressful life events.

This study has limitations in the following aspects. First of all, this study is a cross-sectional study and no causal conclusion can be drawn. Experimental designs can be incorporated into future studies to enhance the causal interpretation of the results. The second limitation is that our subjects are mainly from Northeastern Provinces of China, the problem of appearance anxiety may be more common or have different manifestations in the developed coastal urban cities. Therefore, future research should be conducted in China's first-tier cities (such as Beijing, Shanghai, etc.).

In conclusion, our results revealed the positive relationship between appearance anxiety and social anxiety, and self-compassion can buffer this mechanism. The study raises awareness and has enriched the theory of self-objectification. The results suggest self-compassion training could be part of the treatment for appearance anxiety and social anxiety.

## Data availability statement

The raw data supporting the conclusions of this article will be made available by the corresponding authors, under reasonable request.

## Ethics statement

The studies involving human participants were reviewed and approved by the Ethics Committee of Jilin University. Written informed consent to participate in this study was provided by the participants' legal guardian/next of kin.

## Author contributions

Conception, design of the study, and study supervision: YW and SX. Data collection: SX, HL, XW, and XS. Data quality control: SX, HL, and XW. Manuscript write-up, data analysis, and all figures: YF and JG. Critical comments: YW and AW. All authors contributed to the article and approved the submitted version.
